# Virus Safety of Blood and Plasma Products: From Established Threats to Emerging Uncertainties

**DOI:** 10.3390/pathogens15101006

**Published:** 2026-09-23

**Authors:** Thomas R. Kreil

**Affiliations:** Global Pathogen Safety, Takeda Manufacturing Austria AG, 1221 Vienna, Austria; thomas.kreil@takeda.com

**Keywords:** plasma-derived medicinal products, blood components, viral safety, pathogen reduction, emerging viruses, transfusion safety

## Abstract

Following virus transmissions associated with plasma-derived medicinal products (PDMPs) particularly in the 1980s and 1990s, product safety was substantially improved through the “Safety Tripod”: deferral of higher risk donors, testing of plasma for infectious disease markers, and, quantitatively most importantly, pathogen removal and inactivation steps within their industrial manufacturing processes. As a result, virus transmissions by PDMPs have not been reported for many years, including emerging viruses not even tested for. Blood components for transfusion also rely on donor deferral and donation testing, but unlike PDMPs they do not enjoy a manufacturing process that provides for robust virus reduction. Although sensitive nucleic acid tests have reduced HIV, HBV, and HCV transmission risks to below one in a million, additional screening has been required for emerging or newly recognized viruses such as West Nile virus or hepatitis E virus, yet transfusion-transmitted infections still occur. Advances in virology now indicate that known viruses represent only a small fraction of those in circulation, some of which may pose risks after long incubation periods or in immunocompromised patients. As medical progress, particularly in oncology, increases the population of immune-deficient individuals, more dependable blood and plasma safety margins are needed. Implementing virus reduction across PDMPs and blood components represents a science-based, economically reasonable strategy that should inform future regulatory requirements.

## 1. Introduction

After virus transmissions by plasma-derived medicinal products (PDMPs) were recognized, initially hepatitis B virus (HBV), and more dramatically human immunodeficiency virus (HIV) and hepatitis C virus (HCV) in the 1980s and 1990s [1], their safety margins were greatly and sustainably improved by the implementation of a concept known as the “Safety Tripod”. The concept comprises the deferral of plasma donors who may be at higher risk of carrying an infectious disease, testing for the absence of infectious disease markers in donations and plasma pools, and—quantitatively by far most importantly—validated and robust pathogen removal and inactivation steps, collectively called virus reduction (EU) or clearance (US) steps, embedded into their industrial manufacturing process [2]. Consequently, virus transmissions by PDMPs have not been reported for decades now, and this equally applies to emerging virus concerns that have challenged human health during this time, despite plasma for fractionation not even being tested for their presence.

Donor deferral and donation testing are also used to safeguard blood components for direct transfusion. In contrast to PDMPs though, these products, collected from at least conceptually the same donor veins, do not have an industrial manufacturing process that results in dependable virus reduction. Based on the use of highly sensitive nucleic acid testing (NAT) for the major blood-borne viruses HIV, HBV and HCV, the transmission of these viruses now occurs at a low likelihood of less than one in a million [3]. There has, however, been a need to implement additional tests for the blood supply to detect new viruses as they emerged, were transpositioned, or were realized to be in circulation, such as West Nile virus (WNV) at broad scale in the US [4], and hepatitis E virus (HEV) primarily in Europe [5]. Importantly, despite amended donor selection and extended donation testing, virus transmissions by transfused blood components still do occur.

With advances in molecular virology and bioinformatics, it has become increasingly clear that the currently identified and characterized viruses represent only a minor portion of those in circulation in and around us [6]. And while many of these do not cause acute disease in the general population, they may do so after longer incubation times or in people with immune compromise based on inborn errors, sickness, or drugs. With advances in medicine that allow patients to overcome oncological diseases, for example, yet may render them immune-deficient and thus potentially more vulnerable to infectious diseases, this subsection of the population is expected to grow, and thus it will become even more important to provide for adequate blood and plasma product safety margins, ideally not only against known but also any unknown viruses.

As demonstrated by the difference between the successful pathogen safety record of PDMPs and the continuing challenge of transmissions of a growing range of viruses by blood components, the implementation of effective virus reduction for both these product classes would reflect a science-based approach and an improvement for the virus safety margins of blood components. Traditional requirements around donor deferral and donation testing that are part of the current regulatory framework could then be revisited to, in addition to providing for adequate virus safety margins, include a cost–benefit evaluation and thus maximize access to life-saving therapies for patients by optimizing challenged healthcare economics.

## 2. History and Safety Margins of Current PDMPs

After the development of PDMPs from the middle of the 20th century, which allowed for the treatment of many at the time unserved medical conditions such as polytrauma, immune deficiency and hemophilia, the transmission of viruses as a consequence of pooling the plasma raw material from many thousands of apparently healthy donors was increasingly understood [7]. In response, people with recognized risk factors for certain virus infections, such as men who have sex with men (MSM) or intravenous drug users, were deferred from donating plasma [8]. In addition, as the transmitted viruses were identified and characterized, testing of plasma donations for their absence, or, in the special case of the widely prevalent yet small and physico-chemically more resistant parvovirus B19, to at least dependably limit their load, was implemented.

Testing plasma donors for evidence of virus infection was initially based on the detection of antibodies an infected individual would start to make against the virus, which, however, only became measurable several days or even up to a few weeks after infection. This interval is called window period, and plasma donations collected during this time could contain really high levels of infectious viruses. To prevent pooling of such diagnostic window donations, a mandatory inventory hold period was established for plasma units [9], with the expectation that a subsequent donation from the same donor, now seroconverted for antibody presence, would allow the detection of infection and consequently discard of the seroconverted as well as the earlier window period donation.

The advent of more sensitive NAT changed that situation, as it directly detects the presence of virus nucleic acid itself, and consequently any non-reactive donation at maximum contains virus below the limit of detection. Virus infectivity would, if any, be present several orders of magnitude below the nucleic acid signal, as most virus particles detected by genome presence are not infectious [10]. As a consequence of these scientific improvements, serology testing for antibodies could be replaced by NAT and the inventory hold eliminated. As a science-based and applicable regulatory guidance explains, the risk for virus transmission is calculated from “… *sensitive NAT testing of the manufacturing pool* which *defines a well-controlled upper limit for a potential virus contamination* …, … *the volume of plasma required to produce one vial of product* … and … *virus reduction factor obtained from validation studies* …” [11].

As NAT and virus reduction define the residual risk of virus transmission, the additional value of other, historically implemented measures is less clear, yet they do come at a very substantial cost.

In combination with testing plasma for virus and markers of infection, dedicated virus inactivation and removal processes, such as notably pasteurization from the 1940s and later solvent–detergent (S/D) treatments from 1984 to 1985 [12,13], as well as nanofiltration from the early 1990s [14], were introduced into the manufacturing processes of plasma derivatives. During this technological industry transformation, the last documented virus transmissions occurred through a factor VIII product treated with S/D as the only virus inactivation method, which by its mode of action only inactivates lipid-enveloped viruses—but not the non-lipid-enveloped hepatitis A virus (HAV; the actually transmitted pathogen) [15]. HAV infectivity of the product was confirmed by inoculation into a primate model [16], with the authors’ conclusion that “continued use of these agents seems questionable.”

The deployment of double virus inactivation as required by regulatory guidance [11] finally eliminated virus transmissions by PDMPs.

The general concept referred to as the “Safety Tripod” [2] has stood the test of time effectively, and despite newly emerging virus concerns [1]. Even without changes in donor qualification and virus testing strategy, the virus reduction capacity of manufacturing processes upheld adequate product safety margins. The effectiveness of these reduction steps is validated according to the model virus concept [17], using a range of different viruses and measured on a logarithmic scale to generate information broadly applicable to viruses, thus including even viruses not pathogenic to humans for as far as we know (e.g., anelloviruses and pegiviruses [18]). Over time, the effectiveness of virus reduction steps was confirmed by verification studies against new virus challenges. Of note, during the emergence of WNV in the USA from 1999 [19], implementation of NAT was required by the US FDA for blood components but not for plasma for fractionation [20], as the manufacturing processes’ capability to inactivate the low levels of virus in a plasma manufacturing pool had already been demonstrated, and was fully consistent with expectations based on the model virus concept [21,22]. This arbovirus was, however, transmitted by blood components for transfusion even after the implementation of NAT [23]. Beyond WNV, several other arboviruses were unfortunately also found to be transfusion-transmitted [24], whereas none of them were transmitted by PDMPs.

Verification studies that sought to confirm the effectiveness of virus reduction against a number of other emerging viruses, as observed across recent decades, largely also delivered the expected results, based on our understanding of the respective viruses’ physico-chemical properties and the results of virus reduction studies to support the licensure of PDMPs based on the model virus concept. These included the “bird flu” H5N1 influenza [25], Chikungunya virus [26], hepatitis E virus, which as an added complication occurs as lipid-enveloped or non-lipid-enveloped virus versions [27,28], and most recently the MPOX virus [29]. Experimentally establishing the facts beyond doubt was nevertheless important, to support the regulatory decision-making process as much as to provide adequate reassurance for people who depend on these PDMPs.

## 3. Safety of Blood Components for Transfusion

Blood components for transfusion are collected from carefully selected and thus presumably healthy donors, and donations are tested by a range of assays for viruses or surrogate markers of virus infection. The donor selection process is, however, of limited value for asymptomatic or chronic virus infections that do not visibly impact an infected person’s health [30]. Donor deferrals do, however, even when brief, decrease the likelihood of return donations and thus cause long-term donor loss, particularly among first-time donors [31].

Testing for the absence of some viruses also has limitations, in that a non-reactive test result for virus content does not equate to negativity for virus presence—but may reflect a virus concentration below the limit of detection yet, even with this low virus content, potentially enough to cause infection in the recipient of a transfused unit of product [32]. And, despite the constantly increasing portfolio of tests utilized, only a minor section of the viruses in circulation around us are even tested for. This imbalance can be expected to become more pressing in the context of human mass migration, both in search of relaxation during holidays or peace in response to geopolitical crisis, and global warming [33] which we have seen to transposition virus vectors already [34].

Given the imperfection of both donor selection and donation testing, the transfusion of blood components does—infrequently—still transmit viruses [35].

Virus reduction has also had successes for certain blood components [36,37], with platelets in some geographical regions now generally treated for virus inactivation and other benefits. For red cells, the technical task appears more challenging—with their pigmentation quenching the light needed for photoinactivation and any subtle membrane alteration or oxidative stress compounded across their much longer shelf-life of weeks rather than days for platelets. However, technical solutions have been under development for several years, and while none has yet received general regulatory approval, this may soon be the case [36].

It needs to be emphasized though that virus reduction for blood components can conceptually not be as effective as for PDMPs. Firstly, the virus load contained in one “hot” unit may overrun the inactivation capacity for a blood component, whereas the load of one virus-containing plasma unit would be diluted several thousand-fold into a plasma manufacturing pool before inactivation commences. Consequently, HAV [38] and HEV [39] transmissions by transfusion of virus-inactivated blood components have been described, and for these more resistant viruses, the implementation of an NAT-enforced upper limit of virus load may need to be considered. In addition, the manufacturing processes of PDMPs typically contain two or more mechanistically different virus reduction processes, versus a single virus inactivation step—if any—for blood components.

Not to lose sight of one notable exception for PDMPs, the manufacturing process for Human Serum Albumin (HSA) also only contains a single dedicated virus inactivation step, i.e., pasteurization. This step is, however, complemented by significant virus removal capacity of the upstream plasma fractionation process, which in an early study of the 1970s was estimated to result in 5-6 log_10_, i.e., 100.000 to 1.000.000-fold removal of virus. Any residual virus infectivity was subsequently inactivated by the heat treatment [40].

Sensitive virus testing as well as virus reduction solutions come at a price, a challenge for well-resourced health systems and potentially beyond reach for the public health systems in Low- and Middle-Income Countries (LMICs). In the context of these economic challenges, it may be prudent to revisit traditional transfusion practices, particularly as evidence generated more recently through clinical research seems to indicate that more restrictive patient blood management practices may allow us to reduce red blood cell transfusion rates as well as improve patient outcomes [41]. The implementation of virus reduction processes for blood components may also put into question whether all the currently applied donor selection and donation testing requirements would still carry sufficient additional value, or should be evaluated again to ultimately result in adequately safe as well as affordable and thus available blood products.

## 4. Increasing Understanding of the Virome

Historically, the focus of traditional virology has been on those viruses that cause acute disease in humans or economically important livestock, because of the medical and/or economic impact to society caused. The targeted sampling inherent to this approach though is not effective for the detection of viruses which result in latent infections or may cause disease long after infection.

One illustrative example for an impactful latent infection has recently drawn the attention of the medical community, when infection with Epstein–Barr virus (EBV), which affects up to 90% of the population globally and is widely known as the cause of “kissing disease” of moderate severity, was discovered to also increase the risk of developing multiple sclerosis (MS) by approx. 30-fold, years after initial infection. This association was only revealed by epidemiologic surveillance of more than 10 million young adults on military duty [42], which begs the question about how many more such associations might have been missed up to now.

This and some other understood examples of human disease associated with an earlier virus infection, such as shingles with chickenpox virus or various cancers with papillomaviruses, serve to remind us that an apparently healthy donor of blood or plasma may still carry an infectious disease risk from an earlier exposure.

Viruses that generally cause unspecific and only minor diseases in otherwise healthy people, such as a brief course of fever and some headache as mostly caused by respiratory viruses and several arboviruses, may also not be properly diagnosed and thus remain epidemiologically silent. They may, however, cause severe disease in a smaller subset of the population, e.g., newborns, people of advanced age, or immune-compromised individuals. A recent review of patients treated with B-cell depleting antibody which results in secondary immunodeficiency and subsequently diagnosed with infection by various arboviruses has shown a dramatic 79% of lethal outcomes. As an additional impediment to effective epidemiological surveillance of these cases, 95% of the infections could only be characterized by specialized and not generally available molecular testing, not the more widely used testing for virus antibodies, as affected people could not effectively develop antibodies due to their immune compromise [43].

People with immune deficiency or under immune suppression deserve a particularly vigilant look, beyond care and empathy for their long-term condition, also from a virological perspective, as certain viruses appear to only infect humans with these preexisting conditions, potentially exemplified by a recently described human circovirus [44,45]. For this virus, a small and potentially more resistant virus that can cause high levels of viremia [44], the introduction of NAT to ensure virus loads remain under a reliable upper limit would have been under consideration. Fortunately though, testing manufacturing pools constructed from plasma collected in the US, Europe and Japan generated non-reactive results [46]. Other viruses may persistently replicate in people with immune deficiencies, which can result in the generation of new virus variants that evade immune protection afforded by mono- and polyclonal antibodies, such as recently shown for the pandemic SARS-CoV-2 [47], and the polio [48] and rubella vaccine viruses [49].

Adding to the uncertainty, viruses carried by ticks and mosquitos, at least some of which are already known to cause disease in humans, can be expected to become more widely distributed through translocation of or trade with their wildlife hosts [50], yet they are not systematically monitored for [51]. And while these arboviruses are typically lipid-enveloped, and thus effectively inactivated and removed by PDMP manufacturing processes, not all of them are—the impact of which will be discussed in the following section.

Beyond ticks and mosquitos that are at least understood to be effective virus vectors, there are other, currently less well appreciated sources of viruses that may cause infection in humans. For example, it was recently discovered that the aquatic zoonotic covert mortality nodavirus (CMNV) is associated with an emerging ocular disease in humans [52], apparently also transmitted through consumption of raw and undercooked seafood: another lifestyle-associated infection, perhaps? Adding to the concern, this particular virus is quite small and without a lipid envelope, thus potentially more resistant to inactivation, although the levels of viremia reported up to now were moderate.

As a result of the above-mentioned factors, our understanding of viruses is heavily biased, which also limits the range of agents that can even be identified. For an available technical solution, the fully agnostic molecular tool next-generation sequencing (NGS) could, beyond its current use in medical diagnostics or targeted virus detection, also be considered for broader epidemiological purposes [53]: whether this approach is economically feasible at this time remains to be determined.

On top of this, the restricted growth of viruses in host cell lines represents another challenge, which makes virus isolation and characterization a complex, very specialized and laborious task that requires biocontainment facilities and deep cell culture expertise—both not widely available.

Metagenomics does not face these limitations and based on the agnostic nature of data generated, it now appears that probably less than 1% of all existing viruses have been characterized [6].

Of particular relevance for the plasma-derived medicinal product (PDMP) community, the detection of virus-neutralizing antibodies in immunoglobulin lots (IGs) which contain the plasma donations from thousands of donors provides a uniquely powerful alternative tool to reveal the circulation of viruses. This sero-epidemiology approach has been shown to reveal the rather wide circulation of viruses in plasma donors as a representative subsection of the general population, even when very few or no cases of human infection have been reported. For the WNV emergence in the USA after 1999, neutralizing antibody titers in IGs manufactured from plasma collected in the US were shown to correlate well with the cumulative incidence of past WNV infections [54], essentially a validation of the approach. More importantly, using the same approach for plasma collected in the EU, the circulation of WNV was predicted from the testing of IGs, before any human case of WNV infection had been detected [55]. This very fact is another illustration of the rather limited effectiveness of epidemiological surveillance of human health, also in a well-resourced environment, so long as the medical consequences are relatively minor for the general population.

For a more recent example of how impactful the method can be, after the first description of a human infection with the tick-borne Bourbon virus in the US in 2014, only a handful of additional human infection cases had been reported during more than a decade since [56]. Yet a sero-epidemiological investigation based on testing for neutralizing antibodies in only 40 lots of IGs, which reflects approx. 1.3 million plasma donations, suggests past infection in between 0.06 and 0.5% of the US population [57].

The closely related Oz virus has, for now, been described as causative agent of even fewer cases of severe human infections in Japan and South Korea, yet antibodies to this tick-borne virus can be detected in macaques, wild boars and sika deer, as well as in hunters [58]. Work to support a better understanding of the true range of virus circulation in these Eastern Asian populations is pending.

## 5. Advances in Science and Improvement of Access to PDMPs and Blood Components for Transfusion

Not all the safety measures that have been implemented in the past, and then for the very right reasons, need to be maintained and economically further strain the availability of PDMPs, particularly in Low- and Middle-Income Countries (LMICs). And more recent successes have generated convincing real-world evidence in support of alternative ways to achieve reliable virus safety margins, without the full implementation of all the “Safety Tripod” facets.

Specifically, when WNV emerged in the US in 1999 and became widely endemic there in the early 2000s, the US FDA did require the implementation of NAT for transfusable blood components. They did not, however, and at the time surprisingly for some, require the same measure for plasma for fractionation: as solid virus inactivation and removal for the virus had been verified [21], this additional safeguard was not considered necessary [20]. While this context may be viewed as comforting in light of the increasingly wider circulation of arboviruses such as West Nile, dengue, Zika, Usutu, Powassan or Chikungunya, complacency is still ill advised: the before-mentioned viruses are all lipid-enveloped, and have been shown to [21,59,60], or based on this experimental evidence can be safely assumed to, be effectively inactivated by PDMP manufacturing processes. But not all arboviruses are, with tick-borne Coltiviruses [36] or mosquito-borne Seadornaviruses [61] as examples of non-lipid-enveloped viruses that can infect humans. Should they become a more widely prevalent concern, and viremia possible at high levels, then maybe a NAT-enabled reliable upper limit may have to be implemented. With that though, i.e., reliable virus reduction complemented by—if need be—a test-enforced limit for virus load, any further measures as traditionally utilized may lose importance, and may be reconsidered in the context of affordability, and thus availability, in already challenged healthcare cost structures.

Any potential changes in the set of measures originally deployed to support the pathogen safety margins of blood and plasma products will naturally have to be discussed in detail with the patient organizations affected, as well as the regulatory bodies with oversight authority.

Despite the significant successes of the “Safety Tripod” concept over time, beyond *the mantra of blood safety* it may be *time for a new tune* [62]. Donor deferral, given all the unknown viruses and virus latency that does not allow for any behavioral detection, could be accepted as limited and costly—also for the returning donor base. For viruses that occur at high viremia levels, or are more difficult to inactivate or remove, testing of blood and plasma to provide for a well-controlled and suitably sensitive upper limit would still appear reasonable. And with that, a virus inactivation or removal method should not be overwhelmed and thus provide the expected safety margins. As critically important supplement to these safety measures, it is suggested to provide adequate attention and resources to epidemiology to minimize the risk of any unpleasant surprises.

And if nature and evolution would not provide for sufficiently difficult-to-address challenges to human health, the fresh-off-the-press news at the time of writing that scientists have generated several entirely new and viable viruses using an AI algorithm [63] may—rightfully—trigger some additional risk assessments. For now, the intention is to overcome bacterial resistance to natural phages, which may ultimately offer improved treatment options against multidrug resistant bacteria [64]—for now.

## 6. Conclusions

Despite more than a century of virus discovery, recent molecular methods have revealed that the virome around us has until now been poorly characterized, at best. In addition, epidemiological investigations, some utilizing serology testing at scale using IGs as specimens, have established the somewhat unpleasant fact that many more viruses are in circulation in humans, and animals in close proximity to us, than our medical infrastructure is aware of. Circulation without reported clinical cases of human infection may appear not too worrisome. The argument loses sight though of general epidemiology limitations, i.e., the focus on severe and acute clinical cases—that would inherently not reflect the consequences of infection for those with compromised immune systems, a growing subpopulation, or ones that happen many years after the initial infection, which blurs our ability to detect cause and effect associations. The now available option for humans to deliberately add to environmental virus challenges [63] is not particularly settling in this context.

The proven approach to safeguarding PDMPs even against viruses not known to be in circulation is validated and effective virus inactivation and removal, for particularly resistant viruses that may occur at high infectivity titers in plasma, possibly paired with NAT to establish a reliable upper limit for the virus load that may otherwise challenge and eventually overwhelm the virus reduction capacity of down-stream processing. In this context, it is legitimate to ask the question whether additional donor selection and testing measures carry sufficient value to maintain them.

Similarly, for transfusable blood components, it might be more effective to widely implement virus reduction technologies, rather than continuously changing already complex donor questionnaires and adding new tests for any emerging concerns. At some stage, even historically justified measures might be revisited against the potency of verified virus inactivation capacity, which in combination with a reliably controlled upper limit by, e.g., NAT could provide dependable safety margins without them.

The availability of PDMPs and blood products is a significant economic challenge in many parts of the world, and to reimagine virus safety concepts may help alleviate this restriction without compromising adequate safety margins. Nevertheless, these—in the historical context—delicate changes will have to be preceded by discussions with the relevant stakeholders, including patient organizations as well as regulatory and public policy stakeholder groups.

## Data Availability

No new data were created or analyzed in this study. Data sharing is not applicable to this article.

## Abbreviations

The following abbreviations are used in this manuscript:

AI	Artificial intelligence
CMNV	Covert mortality nodavirus
EBV	Epstein–Barr virus
EMA	European Medicines Agency
EU	European Union
FDA	Food and Drug Administration
HAV	Hepatitis A virus
HBV	Hepatitis B virus
HCV	Hepatitis C virus
HEV	Hepatitis E virus
HIV	Human immunodeficiency virus
HSA	Human serum albumin
IG	Immunoglobulin
LMICs	Low- and middle-income countries
MPOX	Mpox
MS	Multiple sclerosis
MSM	Men who have sex with men
NAT	Nucleic acid testing
NGS	Next-generation sequencing
PDMP	Plasma-derived medicinal product
S/D	Solvent–detergent
US	United States
WNV	West Nile virus
WP	Window period
